# Does treatment assignment influence the prognosis of patients with symptomatic severe aortic stenosis?

**DOI:** 10.1186/1476-7120-13-2

**Published:** 2015-01-09

**Authors:** Giovanni Cioffi, Cesare Tomasi, Andrea Rossi, Stefano Nistri, Luigi Tarantini, Giacomo Faden, Carmine Mazzone, Andrea Di Lenarda, Federica Ettori, Carlo Stefenelli, Pompilio Faggiano

**Affiliations:** Department of Cardiology, Villa Bianca Hospital Trento, Via Piave 78, 38100 Trento, Italy; Section of Occupational Medicine and Industrial Hygiene, University of Brescia, Brescia, Italy; Department of Cardiology, University of Verona, Verona, Italy; CMSR Veneto Medica Altavilla Vicentina|, Altavilla Vicentina, Italy; Department of Cardiology, Ospedale civile Belluno, Belluno, Italy; Cardiology Unit, Spedali Civili Brescia, Brescia, Italy; Cardiovascular Center ASS 1 Trieste, Trieste, Italy

**Keywords:** Aortic stenosis, Aortic valve replacement, TAVI, Balloon aortic valvuloplasty, Prognosis

## Abstract

**Objective:**

Aortic valve replacement (AVR) is the standard therapy in patients with symptomatic aortic stenosis (AS). In high surgical risk patients, alternative therapeutic options to medical treatment (MT) such as trans-catheter aortic valve implantation (TAVI) or balloon aortic valvuloplasty (BAV) have been proposed. In this study we evaluated whether treatment assignment influences *per se* the prognosis of these subjects.

**Patients and methods:**

Criteria for treatment assignment were based on patient’s clinical conditions, Logistic EuroSCORE and other co-morbidities ignored by EuroSCORE. Due to baseline clinical differences between patients with diverse treatment assignment, we used propensity score matching to achieve balance.

**Results:**

368 patients were studied: 141 underwent AVR, 127 TAVI, 49 BAV and 51 MT. 84 events (deaths for all causes) occurred during 14 months of follow-up: 11 AVR (8%), 26 TAVI (20%), 18 MT (35%), 29 BAV group (59%). Traditional Cox analysis identified treatment assignment as independent predictor of events (HR 1.82 [CI 1.10-3.25]) together with lower left ventricular ejection fraction, impaired renal function and history of heart failure. Matched Cox analysis by propensity score confirmed treatment assignment as an independent prognosticator of events (HR 1.90 [CI 1.27-2.85]), and showed similar rate events in TAVI and AVR patients, while it was significantly increased in BAV and MT patients.

**Conclusions:**

Treatment assignment may influence outcome of symptomatic patients with AS.

## Introduction

Suggestions for surgery have been clearly defined for patients with aortic stenosis (AS), and there is a consensus that intervention is mandatory in symptomatic patients [1], whose prognosis radically worsens once valve disease symptoms arise. Although aortic valve replacement (AVR) is the standard therapy, in patients with high surgical risk, alternative therapeutic options to medical treatment (MT) such as trans-catheter aortic valve implantation (TAVI) or balloon aortic valvuloplasty (BAV) have been recently introduced [2]. However, the impact on clinical outcomes is very difficult to assess and compare with conventional AVR mainly because of the clinical heterogeneity of the patients in terms of co-morbidities and frailty [3]. Beside patient’s conditions, the treatment assignment, commonly based on a multi-disciplinary evaluation and supported by several currently used risk scores, might have a clinically relevant effect on the outcomes.

Accordingly, in this study, we analyzed and compared clinical features and mid-term outcomes of a large cohort of patients with symptomatic AS assigned to each treatment (AVR, TAVI, BAV and MT) and assessed whether the treatment assignment *per se* had an influence on the prognosis of these patients.

## Patients and methods

The study population included subjects with symptomatic severe AS (defined as indexed aortic valve area & 0.6 cm^2^/m^2^) consecutively referred to a tertiary hospital. All patients underwent a comprehensive evaluation including clinical history, physical examination, blood sample, electrocardiogram, chest radiogram, echocardiography, computed tomography of the chest, coronary arteriography and aortography. Clinical risk assessment was performed with the Logistic EuroSCORE [4]. Treatment assignment was the result of a consensus of clinical cardiologists, interventional cardiologists and cardiac surgeons shared with each patient. Criteria for treatment assignment were based on patient’s clinical conditions, Logistic EuroSCORE and several other coexisting conditions ignored by EuroSCORE that impeded a standardized approach in place of a made personal consensus stated for each patient. Written informed consent was obtained from all study participants. The Institutional Ethic Committee approved the study protocol, which conforms to the ethical guidelines of the 1975 Declaration of Helsinki.

### Echocardiography

Left ventricular (LV) chamber dimensions and wall thicknesses were measured according the recommendations of the American Society of Echocardiography. Relative wall thickness was calculated as two times the posterior wall thickness/LV diastolic diameter ratio and used as index of LV geometry. Index ≥0.43 was indicative of concentric geometry (the 97.5 percentile in normal population) [5]. LV mass was calculated using the Devereux’s formula [6] and indexed for body surface area. LV volumes and ejection fraction (LVEF) were measured by biplane Simpson’s method. Aortic valve area (AVA) was measured by the standard continuity equation method and indexed for body surface area. Ascending aorta dilation was defined as one or more ascending aorta segment diameter ≥ 40 mm, and classified as mild to moderate if <40 but &50 mm and severe if <50 mm. Porcelain aorta was diagnosed by computed tomography in presence of diffuse plate-like calcification involving the ascending aorta precluding cannulation or cross clamping of the ascending aorta.

### Definition of comorbidities

Kidney disease was defined as glomerular filtration rate (GFR) &60 ml/min/1.73/m^2^ [7]. Chronic obstructive pulmonary disease (COPD) was recognized according to published cut off values (GOLD investigators) [8]. Systemic arterial hypertension was considered when blood pressure <140/90 mmHg or in presence of anti-hypertension medical therapy. Hypercholesterolemia was diagnosed if serum cholesterol was < 200 mg/dl or in presence of pharmacological therapy. We also took into account as co-morbidity the presence of cancer (as history and/or coexistent with AS), we calculated the Charlson score index taken as pointer of burden of disease by summing 19 categories of co-morbidities [9], and the Katz score as specific index of frailty [10].

### Statistical analysis

Data are reported as mean values ±1 SD. Between-group comparisons of categorical and continuous variables were performed by χ^*2*^ test and analysis of variance (ANOVA), respectively. Primary end-point was considered all-cause mortality including in-hospital post-operative death. In order to identify the predictors of all-cause mortality, Log cumulative hazard functions were initially computed by univariate and multivariate Cox proportional hazards analyses, in which all recruited patients were included. Variables which were significantly related to the primary end-point in univariate tests (p & 0.05) were included in the multivariate model. The treatment assignment variable was modeled as categorical. It was categorized in 4 classes and codified as 1 = AVR, 2 = TAVI, 3 = MT and 4 = BAV. No pooling was done among patients who underwent TAVI, MT or BAV. Thus, the HR emerged by matched Cox model for the treatment assignment variable was not referred to a specific class vs the others, but to a significant inter-class difference.

### Estimation of propensity scores and matching

Due to the substantial differences in baseline patients’ features between AVR patients and those assigned to other treatments, we used propensity score matching to achieve balance. The propensity score is the conditional probability of receiving an exposure (e.g. different treatment assignment) given a vector of measured covariates, and can be used to adjust for selection bias when assessing causal effects in observational studies [11]. We estimated propensity scores for treatment assignment for symptomatic AS for all patients using a non-parsimonious multivariable logistic regression model. In that model, the following variables which could potentially influence both the treatment assignment and the clinical outcome were included as covariates: age, Charlson co-morbidity index, Logistic EuroSCORE, history of heart failure, LVEF, COPD, GFR and AVA (measured before the treatment assignment). We then used a Gower’s procedure to match each patient assigned to AVR (considered the gold standard treatment) with another patient who was assigned to an alternative treatment, but who had a very similar propensity score, thus matching 127 AVR patients (90% of the 141 patients who underwent AVR) to 127 patients who were assigned to another treatment: 79 TAVI, 14 BAV and 34 MT. Residual imbalances in baseline covariates between treatment groups after propensity score matching were assessed by estimating absolute standardized differences, expressed as a percentage of the pooled standard deviation. Our propensity score matching reduced absolute standardized differences for all observed covariates below 10% (most were below 5%), demonstrating substantial improvement in covariate balance across the groups. For the comparison of the propensity score matched groups, we used a matched Cox model.

Survival rate was analyzed by the Kaplan-Meier method and survival curves were compared by the log-rank test. A *p* value & 0.05 was considered statistically significant. All analyses were performed using SPSS for Windows version 19.0 (SPSS Inc. Chicago, Illinois, USA).

## Results

### Study population

368 subjects with symptomatic AS (71% had at least an episode of congestive heart failure, 16% of syncope, 26% angina pectoris) were consecutively recruited in the present study from June 2007 to October 2008. Their mean age was 78 ± 10 years, 42% females, mean AVA was 0.32 ± 0.12 cm^2^/m^2^ with a peak trans-aortic peak valve gradient of 80 ± 23 mmHg. The prevalence of hypertension was 75%, kidney disease 54%, chronic atrial fibrillation 33%, diabetes mellitus 25%, COPD 23%, porcelain ascending aorta 3%, history of and coexistent cancer 14% and 4%, respectively. The mean LVEF was 50% ± 8, the mean Logistic EuroSCORE was 25%.

141 patients (38%) underwent AVR (mechanical prosthesis in 100, tissue in 41 patients), 127 (35%) TAVI, 49 (13%) BAV and 51 (14%) were managed by optimized pharmacological treatment (group MT). The main variables of study patients divided according to the treatment assignment are shown and compared in Table 1. Group AVR included the youngest patients, prevalently females with better NYHA functional class and renal function, lower prevalence of history of heart failure, lower pulmonary artery systolic pressure and lower Euroscore than all the other groups. The groups BAV and MT had the worse NYHA functional class and renal function. Mean value of Logistic EuroSCORE was significantly different between the groups ranging from 16% in AVR group to 33% in BAV group (p & 0.001).

**Table 1:** **Main characteristics of the 368 study patients divided according to the therapeutic choice**

	Group AVR 141 pts	Group TAVI 127 pts	Group BAV 49 pts	Group MT 51 pts
• Age (years)	72 ± 10	83 ± 8*	84 ± 9*	81 ± 7*
• Female gender (%)	52	32	49^§^	29*^#^
• Hypertension (%)	77	75	69	73
• Diabetes (%)	27	25	20	25
• Atrial fibrillation (%)	27	39	28	37
• NYHA function class (1–4 scale)	2.3 ± 0.8	2.6 ± 0.6*	2.9 ± 0.8*^§^	2.9 ± 0.8*^§^
• History of heart failure (%)	60	73*	88*^§^	76*
• Chronic obstructive pulmonary disease (%)	16	22	39*^§^	25
• Left bundle branch block (%)	8	17*	20*	16*
• Kidney disease (%)	32	65*	71*	71*
• Glomerular filtration rate (ml/min/1.73)	73 ± 26	54 ± 23*	47 ± 26*^§^	48 ± 28*^§^
• Haemoglobin (g/dl)	11.7 ± 1.8	11.9 ± 1.5	11.8 ± 1.4	11.9 ± 1.6
• Serum total colesterol (mg/dl)	162 ± 45	184 ± 47*	162 ± 29^§^	159 ± 38*
• Charlson comorbidity index	2.1 ± 1.5	3.5 ± 1.7*	3.5 ± 1.7*	3.7 ± 2.0*
• Katz score	2.8 ± 1.7	3.8 ± 1.9*	3.8 ± 1.9*	4.2 ± 2.2*
• EUROSCORE	16 ± 12	28 ± 18*	33 ± 18*	31 ± 20*
• LV relative wall thickness	0.55 ± 0.11	0.55 ± 0.11	0.55 ± 0.14	0.56 ± 0.13
• LV mass (gr/m^2^)	209 ± 56	217 ± 59	541 ± 72*^§^°	207 ± 71^#^
• LV end-diastolic diameter (mm)	50.7 ± 0.8	50.7 ± 0.8	52.3 ± 0.9	50. ± 0.9
• LV end-diastolic volume (ml)	139 ± 69	141 ± 69	155 ± 79	132 ± 67
• LV ejection fraction (%)	53 ± 11	51 ± 12	41 ± 11*^§^°	50 ± 10^#^
• Pulmonary artery systolic pressure (mmHg)	38 ± 11	43 ± 14*	47 ± 15*^§^°	43 ± 12*
• Aortic valve area (cm^2^/m^2^)	0.41 ± 0.10	0.35 ± 0.08*°	0.34 ± 0.12*°	0.42 ± 0.13^§#^
• Trans valve area peak gradient (mmHg)	79 ± 24	86 ± 22*	71 ± 27^§^	76 ± 24^§^
• Biscuspid aortic valve (%)	8	1*	4	6
• Beta-blockers (%)	42	28*	21*	35
• ACE-inhibitors/ARBs (%)	65	67	37*^§^°	65#
• Diuretics (%)	70	83*	95*	75
• Statins (%)	46	53	45	63
• Warfarin (%)	80	60*	55*	45*

ACE = angiotensin-converting enzyme; ARB = angiotensine receptor blockers; AVR = traditional surgical aortic valve replacement; BAV-baloon aortic valvuloplasty; LV = left ventricular; MT = Medical Therapy; HYHA = New York Heart Associations; TAVI = tanscatheter aortic valve implentaions..

p & 0.05 vs AVR = *; vs TAVI = §; vs BAV = #; vs MT = °.

**Figure 1:**
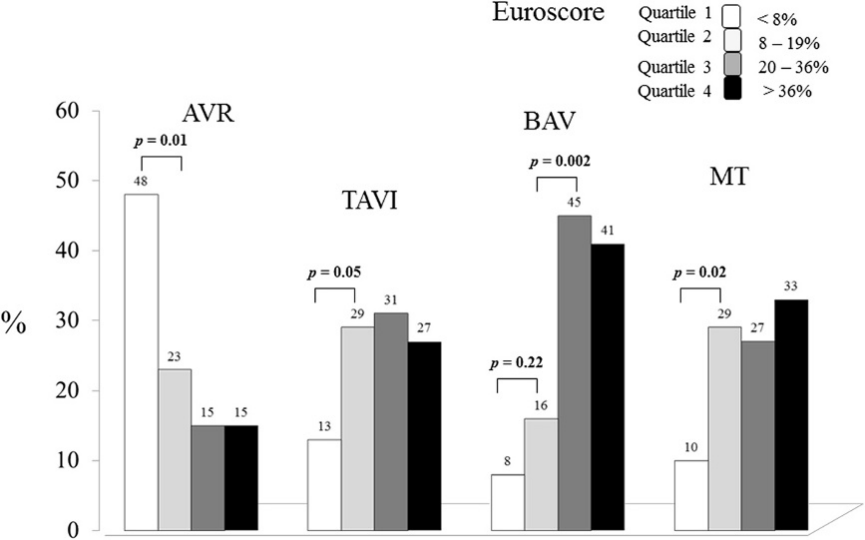
**Distribution of the 368 candidates to the four therapeutic options according to the quartiles of Euroscore.** AVR = aortic valve replacement; TAVI = trans-catheter aortic valve implantation; BAV = balloon aortic valvuloplasty; MT = medical therapy.

The distribution of candidates to the four treatment assignments allocated according to the quartiles of Logistic EuroSCORE is shown in Figure 1. Patients who were not at high risk for AVR according to the EuroSCORE (lower than 20%) were 55 (42%) in the TAVI group, 13 (24%) in the BAV group, and 20 (39%) in the MT group. Reasons for which these patients did not undergo AVR are listed in Table 2. Patients belonging to MT group had all absolute clinical and/or technical contraindication to any procedure of valve replacement or dilatation, independent to Logistic EuroSCORE.

**Table 2:** **Reasons for which aortic valve replacement was excluded as therapeutic option in patients without high Euroscope who underwent treatment allocation to transcatheter valve implantations (TAVI = 55 patients), baloon aortic valvuloplasty (BAV = 13 patients), medicaltherapy (MT = 20 patients)**

Reasons	Number of patients (88)	TAVI ***(55 patients)***	BAV ***(13 patients)***	MT ***(20 patients)***
Advance age (<75 years)	30	16	4	10
Severe chronic obstructive pulmonary disease	14	10	2	2
Porcelain ascending aorta	11	10	0	1
Severe chronic kidney disease	12	8	1	3
Sclerodermia and/or severe arterial pulmonary hypertension	5	3	2	0
Previous coronary-aortic by-pass graft	3	2	0	1
Refuse to undergo surgery	4	1	2	1
Hepatic cirrhosis	3	3	0	0
Thoracic deformation	2	1	1	0
Sever ascending aorta dilatation	1	1	0	0
Cancer	2	0	1	1
Previous stroke	1	0	0	1

### Outcome

**Figure 2:**
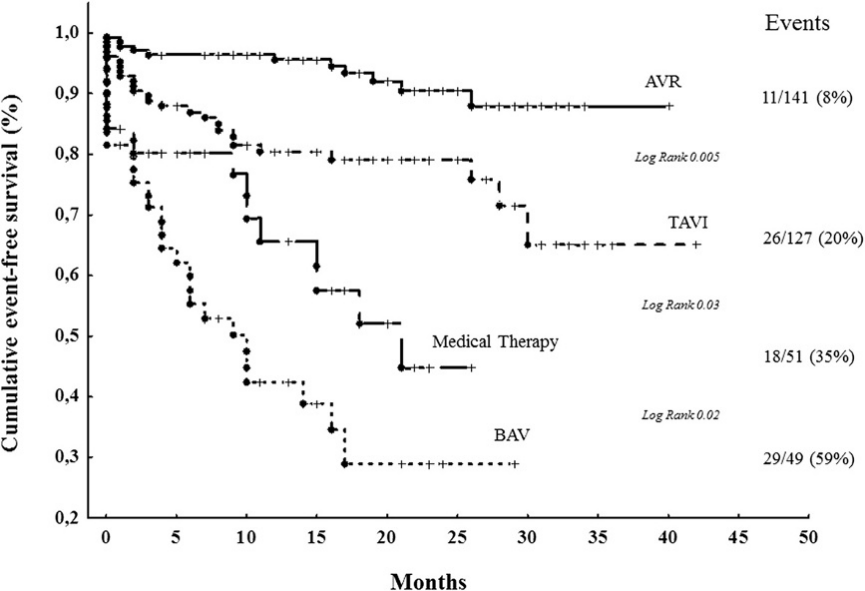
**Event-free survival curves of the full (pre-match) cohort of patients (n = 368) with symptomatic aortic stenosis that underwent AVR, TAVI, BAV and MT (see Figure** 1 **for the abbreviations).**

**Figure 3:**
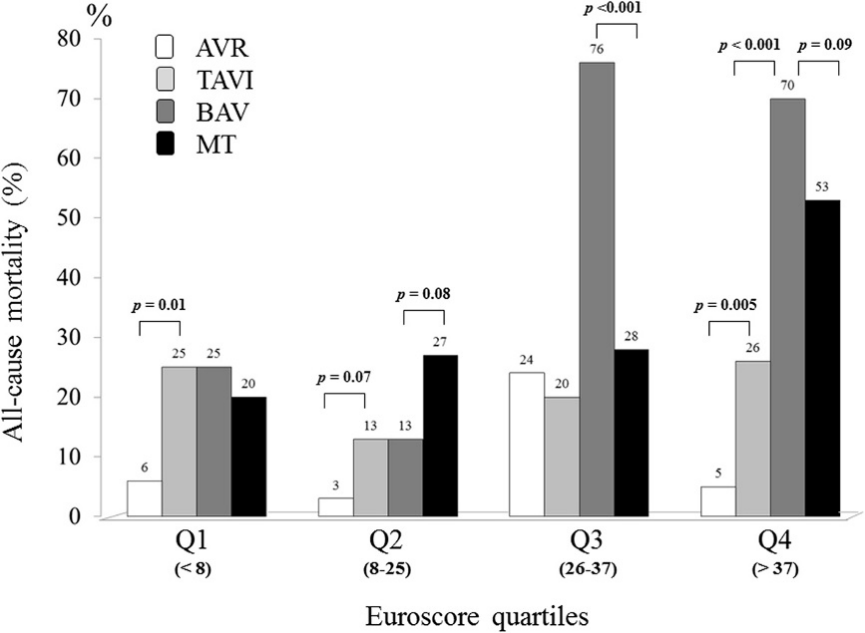
**Incidence of all-cause mortality in the same EuroSCORE quartiles for the four different treatment options.**

Follow-up information was available in all 368 patients. At the end of follow-up (mean 14 ± 9 months, range 0–40), 284 patients (77%) were alive. During this period 84 events (all-cause deaths) (23%) were recorded in this full (pre-match) cohort. The cumulative event-free survival in the whole study population was 85% at 1 year, 69% at 2 years, 50% at 3 years. The primary end-point occurred in 11 of 141 AVR patients (8%), in 26 of 127 TAVI patients (20%), in 18 of 51 MT patients (35%) and in 29 of 49 BAV patients (59%) (overall *p* ≤ 0.03) (Figure 2). Similar trend was observed in the subgroups of patients without high EuroSCORE: all-cause mortality occurred in 5% of AVR group, 20% in TAVI group, 35% in BAV group and 31% in MT group. In comparison with AVR, TAVI patients were older, had higher NYHA functional class and EuroSCORE, lower GFR and AVA. In order to assess the outcome differences related to various treatment choices in a relatively similar status of underlying clinical conditions, we compared the events rate of four different treatment options in the same EuroSCORE quartiles (Figure 3). No difference in mortality rate was found between patients of the four study group in the lowest two quartiles of EuroSCORE, while a significantly higher mortality rate than the other groups was documented in patients undergoing BAV classified in the upper two EuroSCORE quartiles.

Considering the subgroups of patients without high EuroSCORE who did not undergo AVR, all-cause death occurred in 16 out of 88 patients (18%): 9 of 55 TAVI patients (16%), 2 of 13 BAV patients (15%), and 5 of 20 MT patients (25%).

Follow-up was significantly shorter in BAV (9.0 ± 8.1 months) and MT groups (8.1 ± 7.2 months) (*p* = ns between BAV and MT group) than in TAVI (13.9 ± 10.2 months) and AVR (19.2 ± 8.4 months) groups (p & 0.05 between the first two groups and the second ones and between TAVI and AVR) due to the much higher incidence of events in the first two groups. Among patients who died during the follow up, the mean time from enrolment and the clinical event was 10.7 ± 8.2 months in AVR patients, 6.8 ± 5.3 in TAVI patients (*p* & 0.05 vs AVR), 6.3 ± 4.9 in MT patients (*p* & 0.05 vs AVR), and 4.8 ± 3.3 in BAV patients (*p* & 0.05 vs all).

Cardiac aetiology could be identified in 50 of 84 deaths (59.5%). The reasons were listed in Table 3. Cardiac deaths occurred more frequently in the BAV (24 of 29 = 83%) and MT (13 of 18 = 72%) groups than in the AVR (4 of 11 = 36%) and TAVI (9 of 26 = 35%) groups.

**Table 3:** **The reasons of cardiac and non-cardiac deaths occurred in each study group**

Reasons	AVR	***TAVI***	***BAV***	***MT***
***Cardiac deaths (n = 50)***				
Sudden death (n = 28)	2	4	10	12
Cardiogenic shock (n = 11)	-	-	10	1
Chronic pump failure (n = 5)	1	1	3	-
Cardiac tamponade (n = 3)	-	3	-	-
Chronic pump failure (n = 5)	1	1	3	-
Cardiac tamponade (n = 3)	-	3	-	-
Acute massive aortic regurgitation (n = 1)	-	-	1	-
Pulmonary emolism (n = 1)	-	1	-	-
Endocarditis (n = 1)	1	-	-	-
***Non Cardiac deaths (n = 34)***				
Cancer (n = 10)	2	4	2	2
Sepsis (n = 9)	2	6	1	-
Complicated hip fracture (n = 3)	-	3	-	-
Canchexia (n = 2)	-	-	1	1
Hepatic cirrhosis (n = 2)	-	1	-	1
Stroke (n = 1)	-	1	-	-
Unknown (n = 7)	3	2	1	1

### Predictors of adverse outcome

Patients who had an adverse clinical event were older, had a worse functional class and renal function, received less frequently angiotensin-converting enzyme inhibitors/angiotensin receptor blockers (ACEi/ARB) and beta-blockers, had a reduced AVA and LVEF, and had a more frequent history of COPD and heart failure than those who had not (Table 4). Treatment assignment was also associated with adverse events.

**Table 4:** **Variables independently related to adverse events in the total study populations (368 patients): Cox proportional hazard univariate and multivariate analysis**

Variables	Values of patients with vs without events	HR univariate analysis	***CI***	***p***	HR multivariate analysis	***CI***	***p***
LV ejection fraction (%)	44 ± 14 vs 52 ± 13	0.96	0.95-0.98	&0.001	0.98	0.96-0.99	0.003
History of heart failure (%)	87 vs 66	1.65	1.28-2.13	&0.001	2.25	1.16-4.34	0.02
GFR (ml/min/1.73/m^2^)	43 ± 24 vs 63 ± 27	0.98	0.97-0.99	&0.001	0.98	0.97-0.99	0.01
Absolute contraindication to AVR (%)	57 vs 27	3.58	2.16-5.94	&0.001	2.39	1.35-4.22	0.003
Treatment assignment for AS	-	2.01	1.62-2.62	&0.001	1.82	1.10-3.25	&0.001
Age (year)	83 ± 8 vs 77 ± 9	1.02	1.01-1.03	0.004	1.05	0.99-1.09	0.07
NYHA functional class (1–4)	2.8 ± 0.7 vs 2.5 ± 0.07	2.02	1.41-2.90	&0.001	1.05	0.81-1.36	0.69
Charlons co-morbidity index	3.9 ± 1.7 vs 2.7 ± 1.8	1.29	1.18-1.42	&0.001	1.25	0.90-1.57	0.60
Katz score	4.1 ± 1.8 vs 3.1 ± 1.9	1.33	1.11-1.55	&0.001	1.31	0.85-1.62	0.52
Aortic valve area (cm^2^/m^2^)	0.61 ± 0.18 vs 0.69 ± 0.20	1.44	0.45-4.41	0.52	0.18	0.01-2.82	0.22
Peripheral artery disease (%)	10 vs 3	3.63	1.32-9.99	0.01	2.36	0.71-7.84	0.16
COPD (%)	36 vs 18	2.48	1.45-4.25	0.01	1.02	0.36-2.84	0.97
Cancer (%)	8 vs 2	1.90	1.11-3.25	0.02	1.07	0.49-2.33	0.87
EuroSCORE (points)	34 ± 22 vs 22 ± 18	1.03	1.02-1.04	&0.001	0.99	0.98-1.01	0.63
ACEi/ARB (%)	49 vs 66	0.48	0.28-0.81	0.006	0.56	0.31-1.01	0.08
Beta-blockers (%)	22 vs 37	0.48	0.26-0.88	0.02	0.57	0.29-1.15	0.12

AS = aortic stenosis; AVR = aortic valve replacement; COPD = chronic obstructive pulmonary disease; GFR = glomerular filtration rate; LV = left ventricular; NYHA = New York Heart Association.

Consistent with the results of univariate model, Cox proportional hazard analysis included age, AVA, NYHA functional class, GFR, COPD, history of heart failure, LVEF fraction, peripheral artery disease, cancer, EuroSCORE, Charlson comorbidity index, Katz score, ACEi/ARB treatment, beta-blocker treatment and the treatment assignment to the AS. We also built a categorical variable named “absolute contraindication to AVR” (yes vs no) for selecting patients who did not undergo AVR due to anatomic or technical reasons independent to the logistic EuroSCORE value. This variable was forced into the model. This analysis identified the treatment assignment as a strong predictor of adverse outcome independent of lower LVEF, lower GFR, history of heart failure and absolute contraindication to AVR (Table 4).

### Propensity-matched analysis

**Figure 4:**
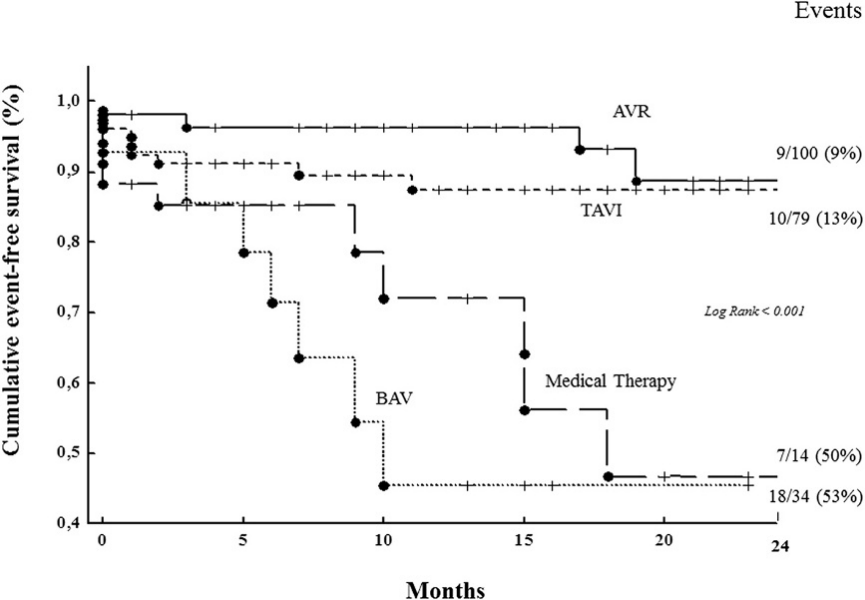
**Event-free survival curves of 254 patients with symptomatic aortic stenosis selected and matched by propensity score analysis who underwent AVR (n = 127), TAVI (n = 79), BAV (n = 14) and MT (n = 34) (see Figure** 1 **for the abbreviations).**

After matching, patients who underwent AVR (n = 127) and those who did not (n = 127) were balanced in all of measured baseline covariates: age 80 ± 6 vs 81 ± 7 years (p = 0.24), Charlson co-morbidity index 3.3 ± 1.5 vs 3.7 ± 1.6 (p = 0.09), Katz score 3.3 ± 1.3 vs 3.6 ± 1.6 (p = 0.11), history of heart failure 70% vs 81% (p = 0.17), EuroSCORE 25 ± 1.5 vs 29 ± 21 points (p = 0.09), LVEF 51% ± 12 vs 50% ± 12 (p = 0.64), COPD 18% vs 25% (p = 0.47), GFR 58 ± 24 vs 56 ± 18 ml/min/1.73 m2 (p = 0.49), AVA 0.39 ± 0.10 vs 0.38 ± 0.10 cm2/m2 (p = 0.33), respectively. All these variables were also similar between patients who underwent TAVI, BAV and MT. The primary end-point occurred in 46 patients: 11 of 127 AVR patients (9%), in 10 of 79 TAVI patients (13%), in 7 of 14 BAV patients (50%) and in 18 of 34 MT patients (53%). The independent association between treatment assignment and mortality for all causes that emerged by the traditional Cox regression analysis adjusted for all baseline covariates remained strong and significant when adjusted for propensity scores (HR, 1.90, 95% CI, 1.27–2.85; p = 0.002). Matched Cox analysis by propensity score showed similar rate events in TAVI and AVR patients, while it was significantly increased in BAV and MT patients (Figure 4).

## Discussion

The main and original finding of our experience is that treatment assignment significantly influences outcome of symptomatic patients with AS independently of the traditional well-known prognosticators. A first consideration deriving from the results of our study is that the clinical features of symptomatic patients with severe AS markedly differ in term of age, functional class, LVEF, co-morbidities, frailty and medical therapy in relation to the treatment assignment. Patients assigned to AVR were younger, had a better clinical presentation and reduced operative risk than patients assigned to the other treatments. This is an expected finding if we consider that AVR represents, as of today, the standard of care for symptomatic patients with severe AS while TAVI, BAV and MT have to be considered as a secondary remedy. In our practice, the main reason for choosing alternative approaches to AVR was the high surgical risk calculated by Logistic EuroSCORE. However, a significant proportion of subjects who had not a high risk for AVR (EuroSCORE lower than 20%) underwent TAVI (42%), BAV (24%) and MT (39%), suggesting other factors contributed to the treatment assignment. This result is in line with previous experiences showing that, in spite of encouraging clinical outcomes even among subjects with high EuroSCORE, up to one-third of eligible patients for AVR do not undergo this procedure [12, 13]. Differently to the other studies, in our practice the presence of severely depressed LVEF did not lead to desist from AVR in any patient. Not even the presence of cancer, hepatic chronic disease and surgical technical difficulties could substantially condition the treatment assignment, which was, instead, strongly influenced by the presence of severe COPD, severe renal disease, porcelain ascending aorta and advanced age. Whereas the first three conditions objectively represent prohibitive obstacles for the surgical team and have a well-known negative impact on clinical outcomes [1, 14], the last one is questionable as reason to preclude AVR [14, 15] for symptomatic severe AS. More recently, Grossi et al. [14] demonstrated that Logistic EuroSCORE greatly over-predicted mortality in the elderly patients, reporting a five-year survival of 72%, unlike suggestions from earlier EuroSCORE analyses. Accordingly, in our scenery, 30 of 88 patients (34%) without high EuroSCORE eligible for AVR have been assigned to an alternative treatment just cause of advanced age, signifying older age as the most important reason for excluding our patients from conventional surgery. Of note, in this large subgroup of 88 patients without high EuroSCORE but perceived as unfitting for AVR, the cumulative incidence of all-cause death was 19%, a percentage not significantly different from that found in the remaining 280 patients (68 deaths = 24%), half of them being subjects with low EuroSCORE who underwent AVR. No difference in the cumulative incidence of all-cause death was found in patients with and without high EuroSCORE assigned to MT or TAVI treatment. However, the most striking point regards patients assigned to BAV treatment, conceived too ill for TAVI. The outcomes of BAV were so poor because the technique resulted ineffective or even dangerous in patients who were very old, with the lowest values of LVEF and GFR and the highest values of pulmonary artery hypertension, NYHA functional class and LV mass. Considering the poor clinical presentation and the large number of co-morbidities of BAV patients, the cumulative incidence of all-cause death was quite low (15%) in the subgroup without high EuroSCORE, while it was 4-fold higher in the whole BAV group, showing an unacceptable event rate of 59%, significantly higher than that found in MT patients. Notably, the large majority of these events (83%) were cardiac deaths. These findings have to be food for thought and critical revision of our therapeutic approach, and are accountable, at least in part, for the dominant and novel result of the present study, which is the demonstration that the treatment assignment *per se* had an independent influence on the prognosis of our patients with symptomatic severe AS.

Considering MT group with reference to the natural history of AS, in our setting we found that TAVI significantly improved survival with a 1-year, with a similar benefit on all-cause mortality to AVR group, when the study population was considered after propensity score matching. Literature data on the comparison between AVR and TAVI are limited and apparently irreconcilable. Piazza et al. [16] found a 30-day mortality significantly higher after TAVI than AVR (9.6% vs 2.3%, respectively). On the contrary, Walther et al. [17] and Descoutures et al. [18] reported similar survival rates between the two groups at 1-year (TAVI 73% vs AVR 69%) and 6-month follow-up (both groups 100%), respectively. Also Wenaweser et al. [3], following patients for a longer period (30 months) found similar rates of all-cause mortality for AVR (22.4%) and TAVI (22.6%) patients. Finally, PARTNERS’s investigators [19] definitively demonstrated in a randomized population of patients with AS who are at high risk for operative complications and death (mean age 84 years, mean EuroSCORE 29%), that surgical AVR and TAVI were associated with similar mortality at 30 days (6.5% vs 3.4%) and 1 year (26.8% vs 24.2%), and produced similar improvements in cardiac symptoms.

According to these results and our finding indicating treatment assignment as independent predictor of all-cause mortality, the opened question is: could some BAV and MT patients be safety referred to AVR, in particular some of them excluded just for advanced age? Such question turns into doubt if we consider the recent results of the SOURCE registry [20], in which mortality was powerfully influenced by the EuroSCORE (below 20% in one-third of partecipants) that emerged as its strongest predictor.

Specific considerations have to be made for the BAV approach. We found an excessive mortality (near two third of candidates) in patients undergoing BAV and classified in the two upper quartiles of EuroSCORE, while mortality rate was similar to the other therapeutic approach in less compromised patients. In line with our data, several authors showed unsatisfactory results when this treatment was proposed alone as palliative therapy [21, 22]. Ben-Dor et al. [21] showed that survival after BAV alone was 50% at 6-month follow up. Tissot et al. [22] found no difference in survival between MT and BAV alone (p = 0.36), while showed similar good mid-term outcomes between the primary TAVI/AVR and bridge BAV to TAVI/AVR (p = 0.08). According to our results, indicating that in BAV patients the adverse clinical events occur early after the procedure (mean time 4.8 months), these authors re-evaluated the patients around one month after BAV for a final decision and were able to successfully replace the aortic valve in 74% of cases (46% TAVI and 28% AVR).

### Study limitations

Our study has some limitation. First, despite the total study population included an appropriate number of subjects (n = 368), the number of patients considered for the comparison of the propensity score matched groups was quite limited in the BAV and MT subgroups, leading to a reduced statistical power for the comparison of treatment groups. Second, we presented a mono-centric clinical experience in which novel aspects on the management of patients with symptomatic severe AS had to be overcome, including the learning curve of new non-surgical approaches. Second, we categorized some co-morbidities (yes vs no) which could be graded for intensity and/or clinical impact (i.e. COPD, stroke). The concern is that treatment assignment more than a really valid predictor of adverse outcome, could represent a measure of EuroSCORE, cause of healthier patients were assigned to AVR procedure. However, we gathered information on the patient’s “comorbidities” (Charlson co-morbidity index), “frailty” (Katz score), and analyzed the absolute contraindications to AVR. These are consistent indexes voicing the charge of co-morbidities and weighting the complexity of patient’s status in terms of physical needs, psychological burdens and risk of surgical failure. By our analysis, it emerged that the amount of co-morbidities and the degree of frailty, which were quite low in AVR patients, were similarly higher in patients who underwent TAVI or BAV or MT and did not influence outcome. On the contrary, a history of heart failure, LV and renal dysfunction accounted for patient’s frailty better than more composite indexes (i.e. Charlson, Katz, EuroSCORE) and predicted long-term mortality as well as the treatment assignment.

### Final considerations and conclusions

In our experience the treatment assignment influences outcome of symptomatic patients with AS, so that criteria on treatment modality for these patients have to be called into question. Alternative treatments to AVR have been recently introduced in clinical practice and, by our results, they seem to be overused. Lacking stringent criteria for each treatment modality and data on long-term follow-up, the new scenery may puzzle cardiologists who have to come to a decision often based on individual experience and emotional feelings more than on solid and standardized protocols and scientific statements. Analyzing our data, it emerged the paradoxical occurrence of an invasive procedure (BAV) which was inferior to MT in terms of all-cause mortality. For most of these patients the choice of BAV in place of MT appears critical considering the clinical characteristics of patients assigned to the invasive approach.

Furthermore, most of the “absolute” contraindications to AVR appear to be “relative” contraindications, so that many candidates to surgery were assigned to alternative treatments and deprived of the safest therapeutic option available up today. Thus, our experience visibly springs from the current pattern of available treatments, the assignment of which to the single individual represents one of the most stimulating challenge for the cardiologists. They have to take into account at the same time prognosis, quality of life, patient’s expectancies, cost/benefit ratios, site selection, center and physician experience and suggestions from the red-hot expert consensus document on TAVI [23]. The results of our study lead to conclude that if there is no serious contraindication (low life expectancy, very high EuroSCORE or serious technical difficulty), the patients should be forced to AVR. Waiting for the clearer therapeutic indications deriving by the results of ongoing clinical trials, this maybe the time of better safe than sorry.
